# Excitation and injury of adult ventricular cardiomyocytes by nano- to millisecond electric shocks

**DOI:** 10.1038/s41598-018-26521-2

**Published:** 2018-05-29

**Authors:** Iurii Semenov, Sergey Grigoryev, Johanna U. Neuber, Christian W. Zemlin, Olga N. Pakhomova, Maura Casciola, Andrei G. Pakhomov

**Affiliations:** 1 Frank Reidy Research Center for Bioelectrics, Old Dominion University, Norfolk, VA 23508 USA; 2 Department of Electrical and Computer Engineering, Old Dominion University, Norfolk, VA 23508 USA

## Abstract

Intense electric shocks of nanosecond (ns) duration can become a new modality for more efficient but safer defibrillation. We extended strength-duration curves for excitation of cardiomyocytes down to 200 ns, and compared electroporative damage by proportionally more intense shocks of different duration. Enzymatically isolated murine, rabbit, and swine adult ventricular cardiomyocytes (VCM) were loaded with a Ca^2+^ indicator Fluo-4 or Fluo-5N and subjected to shocks of increasing amplitude until a Ca^2+^ transient was optically detected. Then, the voltage was increased 5-fold, and the electric cell injury was quantified by the uptake of a membrane permeability marker dye, propidium iodide. We established that: (1) Stimuli down to 200-ns duration can elicit Ca^2+^ transients, although repeated ns shocks often evoke abnormal responses, (2) Stimulation thresholds expectedly increase as the shock duration decreases, similarly for VCMs from different species, (3) Stimulation threshold energy is minimal for the shortest shocks, (4) VCM orientation with respect to the electric field does not affect the threshold for ns shocks, and (5) The shortest shocks cause the least electroporation injury. These findings support further exploration of ns defibrillation, although abnormal response patterns to repetitive ns stimuli are of a concern and require mechanistic analysis.

## Introduction

Research into bioeffects and applications of nanosecond pulsed electric fields (nsPEF) has been steadily expanding during the last decades. Historically, most studies focused on lethal cell damage by nsPEF, induction of apoptosis or necrosis^1–6^, and tumor ablation^7–9^. More recently, the focus has been shifting towards fine mechanisms of nsPEF interaction with living cells and biomembranes^10,11^, as well as excitation and activation of cells and tissues by nsPEF^12–17^. Electrostimulation by nsPEF can exploit unique features, such as direct effects on the endoplasmic reticulum (ER)^18,19^ and non-chemical induction of Ca^2+^ transients^15,16,18,19^ and of phosphoinositol signaling^20,21^ even in cells that express no voltage-gated channels. The stimulation process may involve transient injury (“nanoelectroporation”) to the plasma membrane and intracellular membranous structures^22^.

However, the balance of nanoelectroporation and direct opening of voltage-gated (VG) channels by nsPEF in excitable cells and tissues remains an open debate. The process of opening of VG channels (the translocation of the voltage sensor of the channel and the resulting conformational change) takes as long as hundreds of microseconds^23–25^, so it is not clear how nsPEF stimuli, which are orders of magnitude shorter, cause channel opening. Indeed, a number of studies reported that nanoporation likely is the first step which precedes the response of ion channels: it initiates ion leakage and lasting membrane depolarization, resulting in activation of VG Na^+^ and/or Ca^2+^ channels^16,17^. However, isolated frog sciatic nerves could be excited tens of thousands times by 10-ns PEF, suggesting that no membrane injury is involved^14^. Other studies observed no sign of electroporative damage in nsPEF-stimulated striated muscles^26^, rat embryonic cardiomyocytes^27^, and neurons^28^. A recent study in cultured hippocampal neurons reported that electroporation thresholds for 200-ns pulses were always lower than excitation thresholds; nonetheless, the study concluded that action potentials were not necessarily a result of electroporation^13^.

Among many medical applications of electrostimulation, applying intense electric shocks is the most common life-saving procedure for terminating ventricular fibrillation^29–34^. Excitation of a large or of the entire volume of the myocardium by the shock is essential to halt the propagation of fibrillation front, although the exact mechanisms of defibrillation are not fully understood^34,35^. Modern defibrillators deliver biphasic shocks of millisecond duration^36–39^, but their advantage over monophasic shocks in out-of-hospital cardiac arrest patients is not that clear^30,40^. Since the invention of defibrillation, it was considered desirable to limit the defibrillation energy, to minimize collateral damage to the cardiac tissue^41–43^. Electrical shocks above a critical amplitude damage cells^44,45^, and adverse effects of defibrillation, especially at higher energy levels, may include pain and anxiety, cardiac ectopy, tachycardia, arrhythmia, asystole, re-fibrillation, and increased mortality^31,32,46–51^. The principal mechanism of cell damage is electroporation^31,32,52,53^, and the reduction of pulse duration into nanosecond range could reduce the adverse effects by limiting the size of pores formed^22,27,54–56^. Furthermore, short duration of nsPEF minimizes the electrophoretic component of the transmembrane transport^57^. Compared to longer pulses, nsPEF may minimize the undesired loss and uptake of solutes and reduce the osmotic imbalance, improving cardiomyocytes’ chances of recovery and survival after the electric insult. Other potential benefits of nsPEF include more uniform excitation of myocardium, which reduces the risk of induction of new wavefronts that can reinitiate fibrillation, and defibrillation at lower shock energy^12^. Indeed, we were able to both stimulate and defibrillate Langendorff-perfused rabbit hearts with nanosecond shocks, and the associated defibrillation energy was about an order of magnitude lower than that of monophasic millisecond defibrillation^12^.

The present study continues this work by comparing the excitation efficiency and electric injury by shocks of different duration at the cellular level. In primary ventricular cardiomyocytes from three different species (mouse, pig, and rabbit), we established Ca^2+^ activation thresholds for electric shocks of different duration, from several milliseconds down to 200 ns. Next, the amplitude of the shock was increased proportionally to the excitation threshold for the individual cell, and one or several shocks were applied to electroporate the cell. We found that the shortest shocks were the least damaging, as revealed by reduced uptake of the membrane permeability marker dye, propidium (Pr) iodide. While these data proved a much better safety margin for nsPEF, we also observed higher occurrence of distorted Ca^2+^ transients already at the excitation threshold. Such abnormal transients could be caused by the ER damage or inhibition of VG channels; potential role of such effects for defibrillation remains to be explored.

## Materials and Methods

### Isolation of adult ventricular cardiomyocytes (VCM)

All animal protocols were approved by Old Dominion University Institutional Animal Care and Use Committee. All experiments were performed in accordance with relevant guidelines and regulations. The formulation of solutions and suppliers of chemicals are provided in Table 1, with further details or modifications given in text below.

**Table 1:** Composition of buffers for isolation and experimentation with cardiomyocytes from different animal species.

Components	mouse	rabbit		pig			all animal species		
	Perfusion/Digestion	wash	Perfusion/Digestion	cardioplegia	wash	Perfusion/Digestion	Control	Incubation	Tyrode
NaCl^a^	113	133	125	110	128	133	133.5	133.5	140
KCl^a^	4.7	5	4.7	16	4.7	5	4	4	5.4
MgSO_4_^a^	1.2		1.2				1.2	1.2	
MgCl_2_^a^		2		16	1	2			1.5
CaCl_2_^a^				1.2	1.3	0.01	0.2; 0.5; or 1	1	1
Na_2_HPO_4_^a^	0.6								
NaH_2_PO_4_^a^		1.2			1.2	1.2	1.2	1.2	
KH_2_PO_4_^a^	0.6		1.2						
NaHCO_3_^a^	12			10	20				
KHCO_3_^a^	10								
HEPES^a^	10	10	30			10	10	10	10
Glucose^a^	5.5	10	11.1		11.1	10	11.1	11.1	10
Taurine^a^	30		58.5						
Creatine^a^			24.9						
2,3-Butanedione monoxime^a^	10					10			
Bovine serum albumin^a^			0.1%				0.1%	0.1%	
100x Penicillin/streptomycin^b^								1%	
100x Insulin-transferrin-selenium^c^			1%					1%	
50x MEM Amino Acids^c^			2%						
100x MEM Non-Essential Amino Acid mix^c^			1%						
100× MEM Vitamin solution^c^			1%						

All concentrations are in mM unless different units are given in the table. pH of all buffers was set to 7.4. Perfusion/digestion buffers and pig wash buffer were gassed with 95% O_2_/5% CO_2_ at 37 °C.

Suppliers: ^a^Sigma-Aldrich, St. Louis, MO; ^b^Corning, Corning, NY; ^c^Gibco, Gaithersburg, MD.

#### Isolation of mouse VCM

VCM from 3 to 5 month old DBA/2J female mice were isolated by Langendorff perfusion following protocols by Louch *et al*.^58^ with modifications. Mice were injected i.p. with 0.5 cc heparin diluted in phosphate buffered saline (PBS) to 100 IU/ml and anesthetized by inhalation of 2–4% isoflurane in O_2_. The heart was quickly excised and arrested in ice-cold mouse perfusion buffer (Table 1). Aorta was cannulated and the heart was retrogradely perfused using a two-channel syringe pump (Harvard Apparatus, Cambridge, MA) to maintain a stable flow rate of 3 ml/min. Perfusion solution was heated to 37 °C using a rod in-line heater connected to a TC-344B control unit (Warner Instruments, Hamden, CT); temperature was monitored by a digital thermometer BAT-12 (Physitemp Clifton, NJ). Hearts were perfused for 4 min with the perfusion buffer and then for 8 min with digestion buffer (same formulation, but supplemented with 0.1 mg/ml Liberase TM (cat.# 05401127001, Roche, Switzerland) and 12.5 μM CaCl_2_). Next, heart was taken off of the cannula, placed in a 35-mm culture dish with 3 ml of the digestion buffer and moved to a sterile laminar flow hood. Atria were removed, and ventricles were pulled apart with forceps, minced, and then gently triturated with a transfer pipette for 5 min. VCM suspension was filtered through a 100 µm cell strainer into a 50-ml tube and digestion was halted by adding 3 ml of perfusion buffer with 2 mg/ml of BSA fraction V and 12.5 µM CaCl_2_. Cells were left to settle down for 15 min, and the supernatant was replaced with 10 ml of perfusion buffer with 1 mg/ml of BSA fraction V and 12.5 µM CaCl_2_. Next, Ca^2+^ concentration was increased in several steps. First, two aliquots of 50 µl of 10 mM CaCl_2_ each were added to the tube with cells with a 4-min interval. In 7–8 min after the second addition, supernatant was removed and replaced with 10 ml of control buffer with 200 µM CaCl_2_. This procedure was repeated two more times to raise CaCl_2_ concentration to 500 and 1,000 µM, with the same time intervals. Cells were seeded on laminin-coated 10 mm glass cover slips, and in 3 hours the medium was replaced with the incubation buffer. Cell were kept at room temperature and typically used in experiments within 48 hr.

#### Isolation of rabbit VCM

Female New Zealand white rabbits weighing 2–3 kg were injected with sodium heparin (1500 units/kg) in the ear vein 10 min prior to euthanasia. Rabbits were anesthetized with isoflurane (3–5% in 100% O_2_) in an induction chamber. VCM isolation procedures followed on-line instructions by S. C. Armstrong, http://www.usouthal.edu/ishr/help/myocytes/rabbitmyocytes.htm, with modifications. The chest cavity of the anesthetized rabbit was opened, the heart rapidly excised and perfused with a syringe in a retrograde Langendorff mode with ice-cold wash buffer (Table 1). Next, the heart was moved to a Langendorff apparatus and perfused for 5 min with perfusion buffer (PB) gassed with 95% O_2_ 5% CO_2_ at 37 °C. The solution was switched to a digestion buffer (PB supplemented with 200 U/ml of Type II collagenase (Worthington, Lakewood, NJ)) and continued for about 40 min, at 25–40 ml/min in a recirculating fashion, until the heart became pale and soft to touch. Left ventricle was cut out and minced in 60 ml of perfusate. Cells were dispersed by triturating with a plastic transfer pipette with a cut-off tip for 10 minutes at room temperature. Cell suspension was filtered through a 500-μm nylon mesh into three 50-ml tubes (20 ml per tube), and an equal amount of PB with 0.2% BSA was added to each tube. Cells were left to settle for 15–30 min, supernatant was removed and replaced with 40 ml of PB without collagenase. 200 μl of 10 mM CaCl_2_ was added to each tube, cells were gently mixed by inverting tubes upside down several times, and left for 8 min. Another 200 μl aliquote of 10 mM CaCl_2_ was added, mixed, and cells were allowed to settle for 20 min. The next steps of incremental calcium addition, seeding, and incubation were the same as described above for mouse VCM.

#### Isolation of pig VCM

Adult Yorkshire cross domestic pigs weighing 55–60 kg were used for an approved animal protocol unrelated to this study, with an add-on protocol for myocardial tissue collection. Animals were sedated with an oral dose of 6 mg/kg diazepam, followed by an i.v. dose of 20 mg/kg ketamine and 0.5 mg/kg midazolam. The animal was intubated with #5–8 endotracheal tube and anesthesia was sustained with 2–3.5% isoflurane.

VCM isolation procedures followed protocols of Skuse^59^ with modifications. Sternum was cut open, and the heart was removed, cannulated, and perfused with 2 l of ice cold cardioplegia buffer. Next the heart was perfused with 37 °C wash buffer, the apex of the heart was removed and cut in several pieces. Each piece was placed in a 6-well plate filled with PB, rinsed for 10–15 s in each well, and minced in the last well. Tissue pieces were transferred into several 50-ml tubes with 40 ml of 37 °C PB supplemented with 250 U/mL collagenase type II. The tubes were placed on an orbital shaker (200 rpm) and kept at 37 °C. In 15–30 min, a protease inhibitor cocktail of 10 μM Leupeptin (Santa Cruz Biotechnology, Dallas TX), 1 mM Pepstatin, and 1 mM Benzamidine (both from Sigma-Aldrich, St. Louis, MO) was added to spare collagenase activity but block most other proteases which are typically present in commercial collagenase supplies. Tubes were returned to shaker for 30–40 minutes; then approximately 20 ml of the solution with tissue pieces were transferred into a 100-mm Petri dish. Tissue was pulled apart with forceps, and triturated for up to 10 min with a plastic transfer pipette with the tip cut. Cell suspension was filtered through a 500 µm mesh and 20 ml of PB supplemented with 0.2% BSA was added. Cells were allowed to settle for 20 min, and the supernatant was replaced with 20 ml of the PB with 0.1% BSA. In 8 min, 100 μl of 10 mM CaCl_2_ was added to each tube, and cells were gently mixed. In 8 min, the same aliquot was added again, mixed, and cells were left to settle down for 20 min. The next steps of incremental calcium addition, seeding, and incubation were the same as described above for mouse VCM.

### Stimulation and electroporation by electric pulses

**Figure 1:**
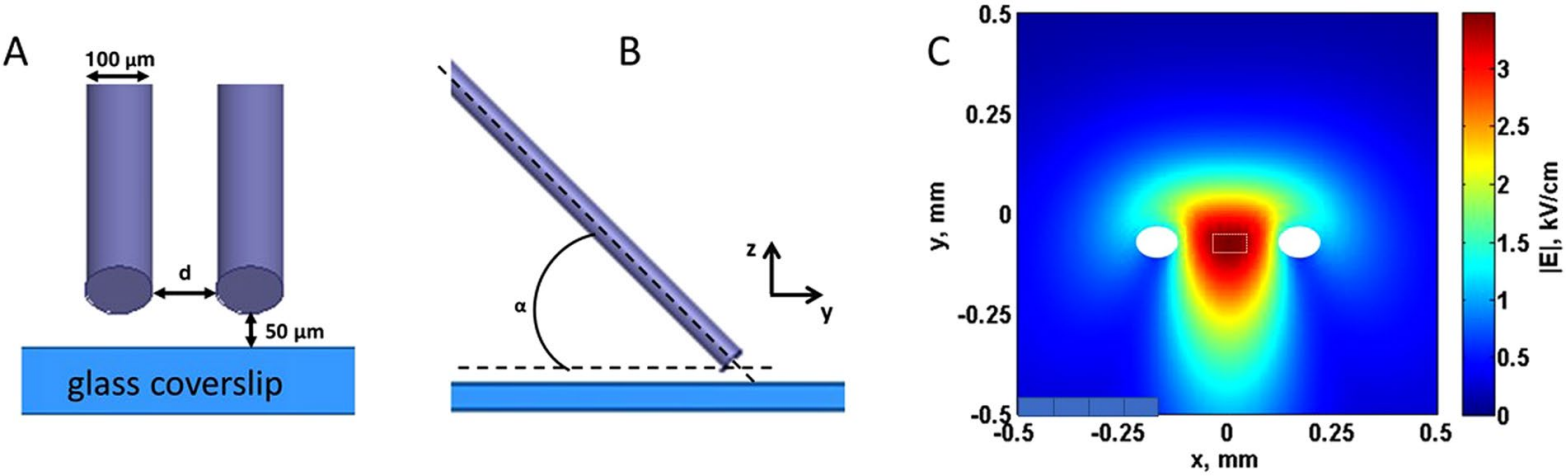
A diagram of the single cell stimulation set-up (**A**,**B**) and numerical simulation of the electric field distribution (**C**). Tungsten electrodes, 100 μm in diameter, were positioned precisely at 50 μm above the glass coverslip with seeded cells (not shown). The angle to the coverslip was about 30°. A and B are the front and side views of the electrode position. The gap **d** between the electrodes varied in different sets of experiments from 170 to 300 μm, and the electric field between the electrodes was re-calculated for each gap distance. In panel C, the electric field values are calculated for 100 V applied to the electrodes with a gap of 170 μm. The position of electrode tips is denoted by white ovals. Electric field values reported in this paper are the average values for a 40 × 90 µm region in the middle of the gap between electrode tips (white dotted line rectangle), at 10 µm above the coverslip surface.

Field stimulation and electroporation of individual selected cells on a microscope stage were described in detail previously^54,60^. A pair of tungsten rod electrodes (100 μm diameter, 170- to 300-μm gap) was connected to either a MOSFET-based generator to deliver nsPEF stimuli, or to a Grass S88 stimulator (Grass Instrument, Quincy, MA). Using an MPC-255 robotic manipulator (Sutter, Novato, CA), the electrodes were positioned within the microscope field of vision so that the selected cell was centered between the tips of the electrodes (either perpendicular or parallel to the electric field); then the electrodes were lifted to precisely 50 µm above the coverslip surface (Fig. 1).

To produce nanosecond pulses of a predetermined duration (down to 200 ns) and amplitude, a capacitor of a custom-made nsPEF generator was fully charged to a desired voltage from a high-voltage DC power supply. The capacitor was turned on and off by a power MOSFET switch (IXYS, IXFB38N100Q2) for a given period of time, controlled with a digital delay generator (model 577-8 C, Berkeley Nucleonics Corporation, San Rafael, CA). In turn, the delay generator was triggered and synchronized with image acquisitions by a TTL pulse protocol using Digidata 1440 A board and Clampex v. 10.2 software (Molecular Devices, Sunnyvale, CA). To produce micro- and millisecond range pulses, TTL trigger was sent to the Grass stimulator instead. The pulse shapes and amplitudes were monitored with a TDS 3052 oscilloscope (Tektronix, Beaverton, OR).

The electric field applied was determined as described previously^13^ by 3D numerical simulations using a commercial finite element solver COMSOL Multiphysics, Release 5.0 (COMSOL Inc., Stockholm, Sweden). Briefly, in the model, two parallel rod electrodes (1 mm long, 100 μm diameter, 170- to 300-μm gap, stainless steel) were inclined at 35 °, positioned 50 µm above the glass cover slip (100 μm thick, conductivity 0 S/m, relative permittivity 3.78), and immersed in physiological solution (1 mm deep, conductivity 1.4 S/m, relative permittivity 76). The model was enclosed in a sphere of air with radius of 3 mm. The whole domain of simulation was meshed resulting in a total of 1,577,538 tetrahedral elements, with a minimum size of 1.2 µm and maximum size of 210 µm. Quadratic elements were used throughout the solution domain, giving 2 × 10^6^ degrees of freedom. The Electric Currents interface was used to solve Maxwell’s equations under the assumption of steady-state conditions. Electric field values reported below are the average values for a region of 40 × 90 µm in the middle of the gap between electrode tips, at 10 µm above the coverslip surface (Fig. 1C). For the electrodes with a 170- or 300-µm gap, the coefficient of variation, calculated as ratio of the standard deviation over the mean value of the electric field, was 5.7% and 3.9%, respectively. Although cardiomyocytes are large cells and their portions could extend beyond this area of practically uniform electric field and experience lower field intensities, the excitation thresholds and the electroporative damage were both determined by the highest electric field imposed on cells, i.e., by the field in the 40 × 90 µm central region.

Uniformly for all types of experiments, we tested two pulse durations from nanosecond range (200 and 800 ns), one or two pulse durations from microsecond range (usually 200 µs; sometimes supplemented with 10 or 50 µs, see below) and one pulse duration from ms range (2 or 4 ms). In experiments with VCM permeabilization by trains of 20 pulses, we could not use any data for pulse duration in excess of 10 µs due to intense bubble formation at the stimulating electrode. For fast measurements which did not require long observation (shapes of Ca^2+^ transients) we added extra datapoints at intermediate pulse durations of 400 ns, 2 and 5 µs. The amplitude of pulses was set either at the stimulation threshold or at 5x the threshold, as indicated in text below.

The maximal theoretically possible (adiabatic) heating caused by pulses of different duration was calculated from the absorbed dose, as described previously^61,62^. Out of all nsPEF treatments tested in these study, the largest adiabatic heating (for a train of 20, 200-ns, 12.2 kV/cm pulses) equaled only 2 °C, and in reality it was even less due to heat dissipation. Thermal effects from single stimuli at any tested pulse durations and intensities did not exceed 0.1 °C.

### Optical Detection of Ca^2+^ transients and Pr uptake

Cytosolic Ca^2+^ was monitored by fluorescence imaging with Fluo-4 (Invitrogen, Carlsbad, CA). Cells were loaded with the dye by incubation for 15 min in Tyrode solution (Table 1) containing 5 μM of Fluo-4/AM and 0.02% of Pluronic F-127 (Life Technologies, Grand Island, NY), in the dark at room temperature. The coverslips were rinsed twice and then left for 15 min in the physiological solution before being transferred into a glass-bottomed chamber (Warner Instruments, Hamden, CT) mounted on an Olympus IX81 inverted microscope equipped with an FV1000 confocal laser scanning system (Olympus America, Center Valley, PA). The chamber was filled with Tyrode buffer supplemented with 10 or 20 µM blebbistatin to prevent cell movement artifacts, and with 5 or 10 µg/ml of an established membrane permeability marker dye, Pr iodide. This dye is essentially non-fluorescent when in the chamber solution, but once Pr cation enters the cell, the emission increases profoundly upon its binding to intracellular nucleic acids.

**Figure 2:**
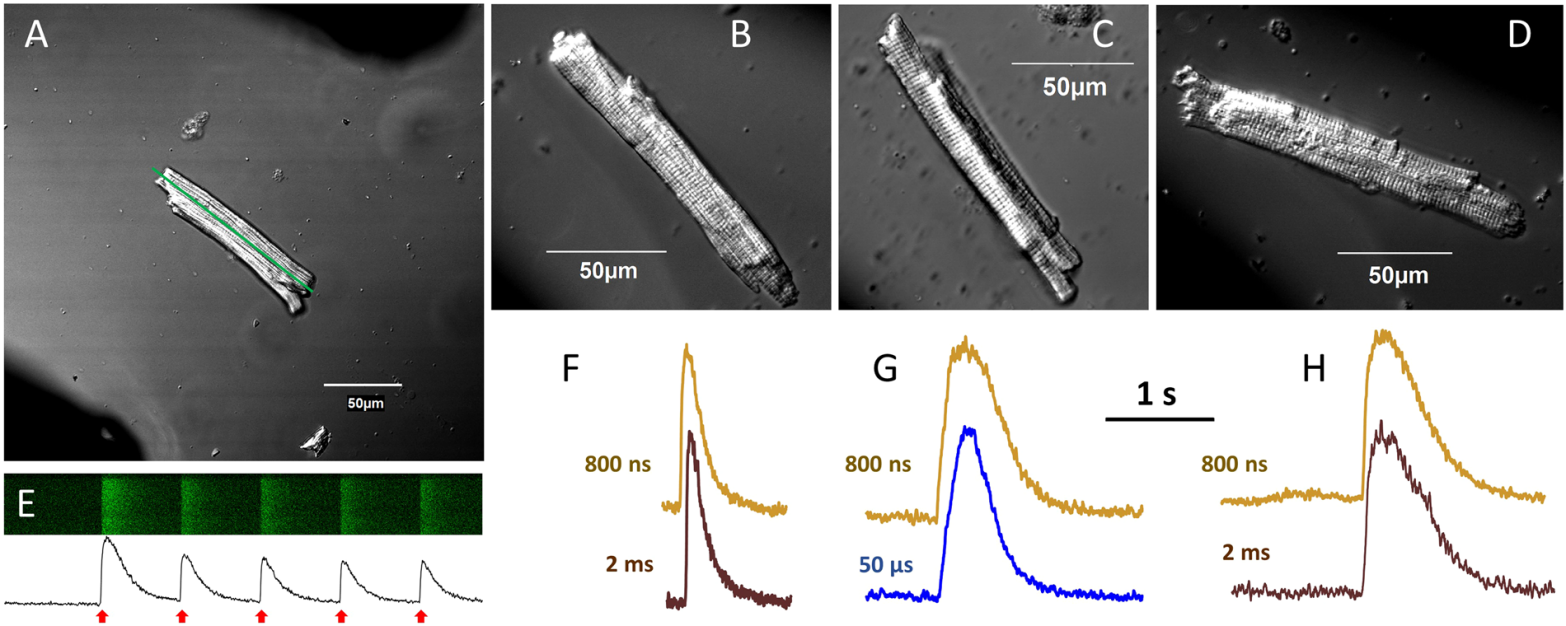
Recording of Ca^2+^ transients in isolated ventricular cardiomyocytes from mouse (**A**,**B**,**E**,**F**), rabbit (**C**,**G**), and pig (**D**,**H**). In VCM images (**A**–**D**), dark areas in the diagonal corners show the location of stimulating electrodes (the inter-electrode distance could vary from 170 to 300 μm). In A, the line along the axis of the cell is the region for line scan of Fluo-4 fluorescence (**E**, top). The fluorescence intensity plotted versus time (**E**, bottom) reveals Ca^2+^ transients elicited, in this example, by 5, 200-μs stimuli applied with 1-s intervals (red arrows). Panels F, G, and H show typical Ca^2+^ transients recorded from mouse, rabbit, and pig VCM, respectively. Pulse duration is indicated next to the traces.

All experiments were performed at room temperature. Images were taken with a 40X, NA 0.95 dry objective. Fluo-4 fluorescence was detected in a line scan mode (usually, 2 ms/scan), with the line drawn approximately through the center of the cell parallel to is long axis (Fig. 2A). Fluo-4 was excited with a blue laser (488 nm) and the emission of the dye was detected between 505 and 605 nm. Image acquisition was synchronized with nsPEF delivery by a TTL pulse protocol from pClamp software via a Digidata 1322 A output (Molecular Devices, Sunnyvale, CA). The acquisition typically continued for 6 s and 5 stimuli were applied with 1-s intervals.

In some sets of experiments, a low-affinity Ca^2+^ indicator Fluo-5N (Thermo Fisher Scientific, Waltham, MA) was used instead, to enable a more faithful recording of the shape of Ca^2+^ transients^63^. The dye was loaded into cells according to supplier’s recommendations. Within limits of this study, we have not observed any consistent difference from Fluo-4 data, and results were analyzed together.

PI emission was excited with a 543 nm laser and detected in the wavelength range 560–660 nm or 655–755 nm. Cell images were taken once in 10 s for 5 min, with the first 3 images acquired before nsPEF delivery, which was done at 27 s from the start of recording.

The sensitivity of fluorescence detector was kept constant within each series of experiments, but could be adjusted for different series, in order to maximize the dynamic range of the detector while avoiding its saturation. Therefore, the arbitrary units (a.u.) of fluorescence shown in different figures are not necessarily comparable.

Images were processed and quantified using MetaMorph Advanced v.7.7.0.0 (Molecular Devices). Data are presented as mean ± s.e. Statistical analyses were performed using a two-tailed *t*-test where p < 0.05 was considered statistically significant.

### Data availability

The datasets generated during and/or analyzed during the current study are available from the corresponding author on reasonable request.

## Results and Discussion

### Nanosecond pulses can evoke Ca^2+^ transients similarly to conventional stimuli

Once the coverslip with VCM attached was placed on the microscope stage, the stage was moved to search for a single (not obscured by other cells), rod-shaped VCM without any apparent lesions. Once a suitable VCM was located, stimulation electrodes were moved into the work position, so that the VCM was in the middle of the gap between the electrodes, with its long axis approximately parallel to the electrodes and perpendicular to the electric field (within +/−20–30° angle, Fig. 2A–D). Applying single or repetitive stimuli caused characteristic patterns of line scan detection of Fluo-4 dye fluorescence, with the intensity peaks corresponding to Ca^2+^ transients (Fig. 2E). Preliminary experiments established that sub-microsecond pulses can evoke Ca^2+^ transients in VCM of all three tested species, and the shape of the transients appeared to depend on the animal species (shorter transients in VCM from the species with a faster heartbeat) rather than on the stimulus duration (Fig. 2F–H).

**Figure 3:**
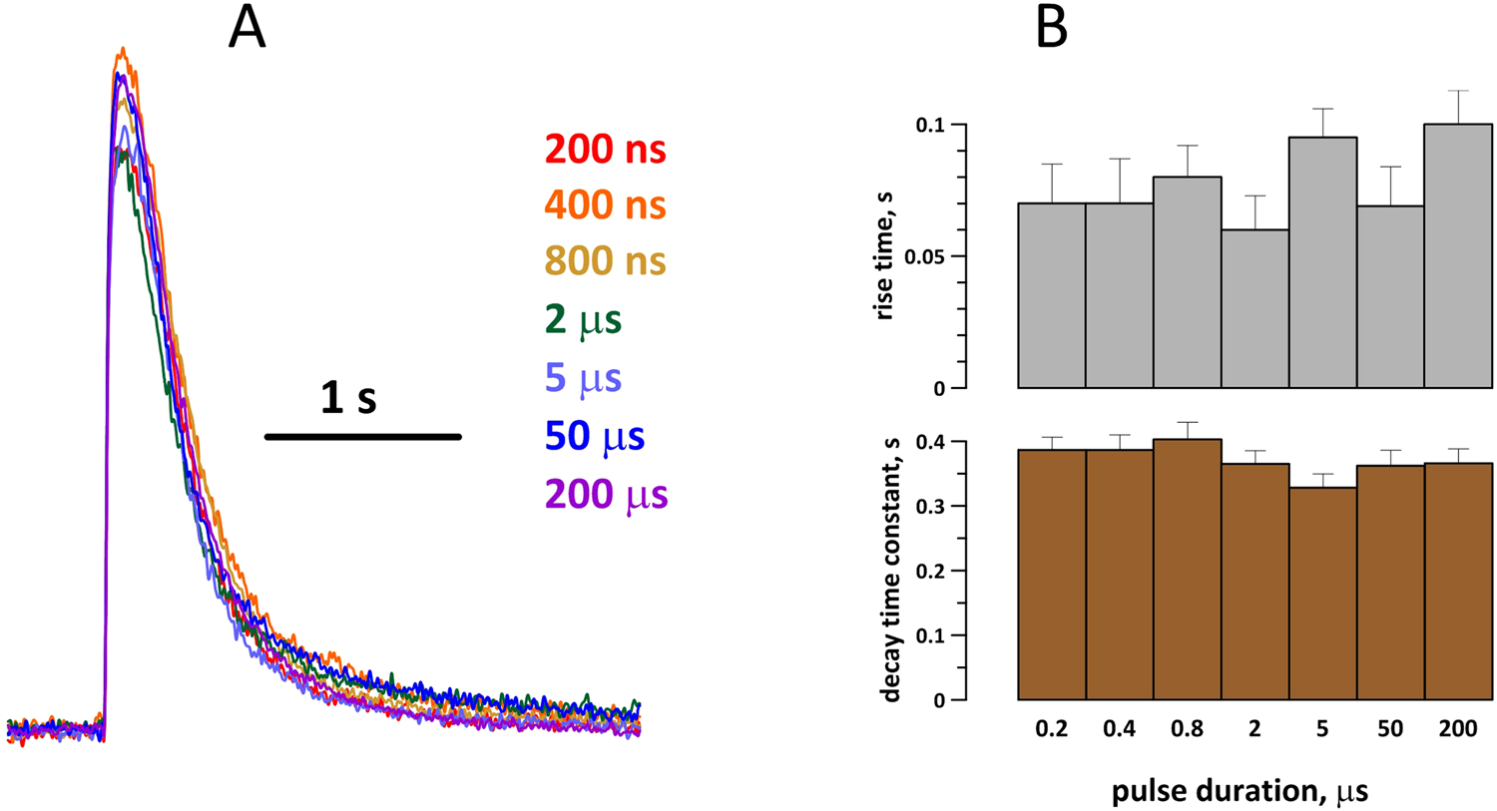
Stimulus duration has no effect on the shape of Ca^2+^ transients. (**A**) overlapped traces of transients as averaged from at least 5 mouse VCM. The cells were stimulated at the excitation threshold, by pulses ranging in duration from 200 ns to 200 μs (color-coded). (**B**) The analysis of the average rise time and decay time constant of Ca^2+^ transients in individual stimulated cells (mean +/− s.e.) shows no statistically significant impact of pulse duration.

A detailed analysis of the time course of Ca^2+^ transients evoked by different stimuli was performed in mouse VCM (Fig. 3). Transients evoked by stimuli of 7 different durations, in a minimum of 5 cells for each stimulus duration, were averaged and plotted together (Fig. 3A), and also were quantified in individual cells for statistical comparison (Fig. 3B). Any transients with “distorted” shape (see below) were not considered for this analysis. Measured variables were the rise time and the decay time constant (by fitting with a single-exponential function) of each individual transient; Fig. 3B does not show any statistically significant differences between Ca^2+^ transients evoked by stimuli of different durations.

### Extension of strength-duration curves into nanosecond range

**Figure 4:**
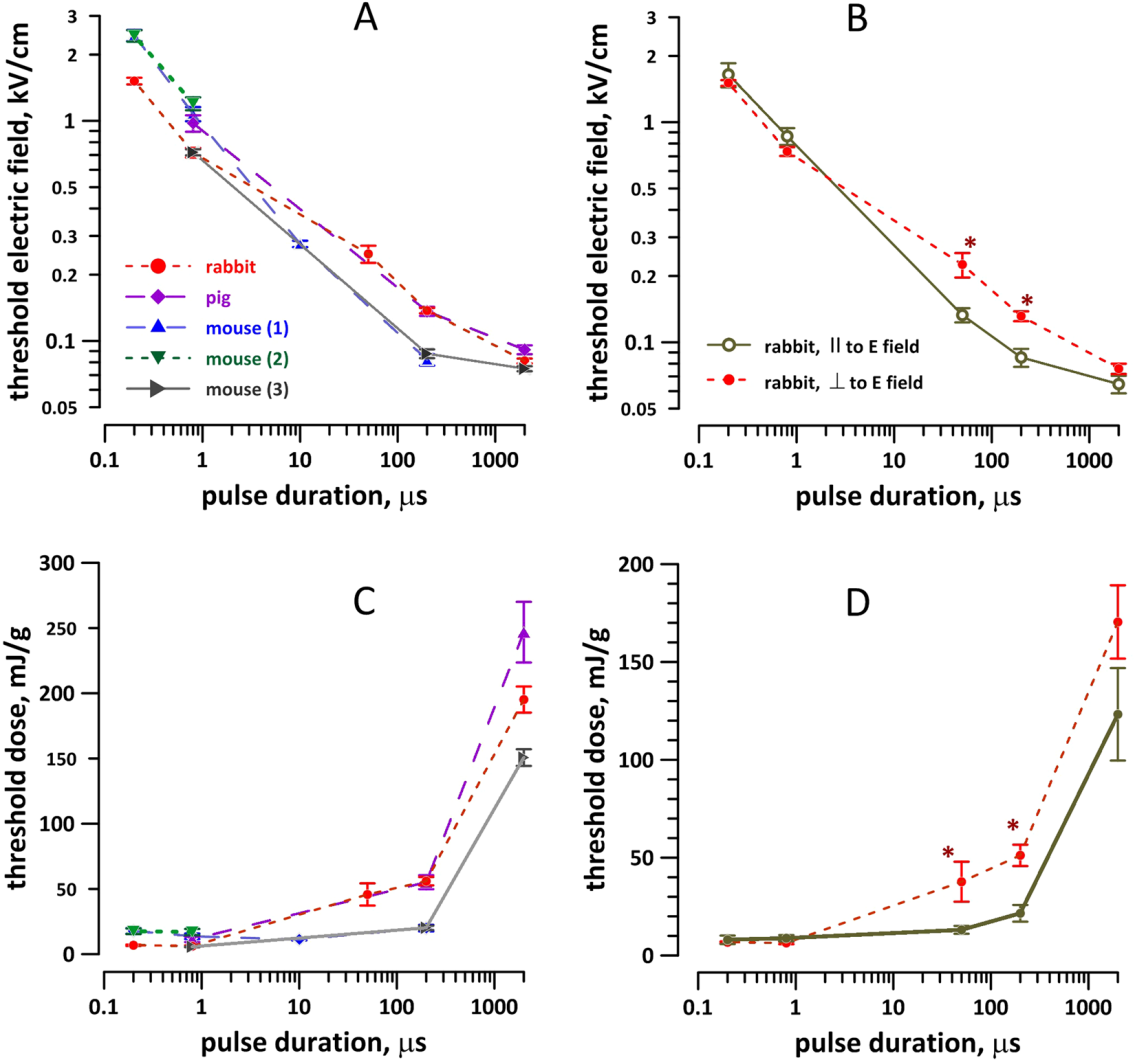
Effect of stimulus duration (**A**,**C**) and cell orientation in the electric field (**B**,**D**) on Ca^2+^ activation thresholds in adult VCM from different species. The thresholds are expressed as the electric field (**A**,**B**) or the respective dose (**C**,**D**). For experiments in panels A and C, all cells were oriented perpendicular to the electric field. Each datapoint is the mean +/− s. e. for 6 to 15 cells; each cell was probed with only one stimulus duration. Three groups of murine VCM (**A**,**B**) are from different sets of experiments separated by several months; they are shown separately to better illustrate the reproducibility of measurements. *Significant effect of cell orientation (**B**,**D**), p < 0.01, 2-tailed t-test.

Stimulation thresholds for pulses from 200 ns to 2 ms duration were established in several independent series of experiments, performed over a time period of about a year. In a typical experiment, we applied trains of 5 stimuli with 1-s interval (like in Fig. 2E). Voltage delivered to the stimulating electrodes was raised in 10–15% increments, starting from presumed sub-threshold levels, and until a Ca^2+^ response was observed. The corresponding electric field value was noted as a stimulation threshold for the specific cell. The threshold data for over 200 individual cells positioned perpendicular to the electric field are summarized in Fig. 4A; the thresholds for parallel and perpendicular orientations with respect to the electric field are compared in Fig. 4B. The response thresholds expectedly increased as the pulse duration decreased, similarly for VCM from mouse, rabbit, and pig. The data showed excellent reproducibility from one set of experiments to another, and less than 2-fold difference between the species. Of note, the data for 2-ms pulses should be taken with caution, because of bubble formation at the cathode and possible reduction of the electric field reaching the cell.

Orienting the VCM along the electric field lines lowered the threshold for “long” 50- and 200-µs pulses 1.5–1.7 times, which is close to a 2-fold reduction reported by other authors for a different stimulation set-up^64^. However, cell orientation did not affect the threshold for 200- or 800-ns pulses (Fig. 4B). Indeed, the potential induced on cell membrane by an external electric field increases linearly with increasing the cell dimension along the electric field lines (Maxwell-Wagner polarization), so orienting the cell’s long axis along the field lines induces the threshold transmembrane potential at a lower external electric field. The lack of such dependence for nsPEF stimuli indicates that the membrane did not get fully charged within the duration of the stimulus, so the cell dimension along the electric field lines had little or no impact. The independence of the stimulation threshold from cell orientation may translate in a more uniform excitation of heart tissue *in vivo*, which would be beneficial for defibrillation.

Interestingly, nsPEF stimulation also required lower energy to excite VCM, for all tested species and for both VCM orientations (Fig. 4C,D). Since damaging effects of defibrillation correlate with the energy of the shock, lowering the energy by reducing the pulse duration may also reduce the undesired side effects of defibrillation.

### Repetitive nsPEF stimuli evoke distorted Ca^2+^ transients

The data presented above in Figs 2 and 3 suggest that nsPEF induce Ca^2+^ transients similarly to conventional stimuli, by engaging the same well-known physiological mechanisms. Thus far, these data provided no indication of differences in the opening of VG channels, Ca^2+^ mobilization from the ER, or its clearance from the cytosol after nsPEF versus conventional stimuli. The strength-duration curves in the nanosecond range continued the same pattern as with longer pulses (Fig. 4), which serves as an additional indication of the similarity of excitation mechanisms^26^.

**Figure 5:**
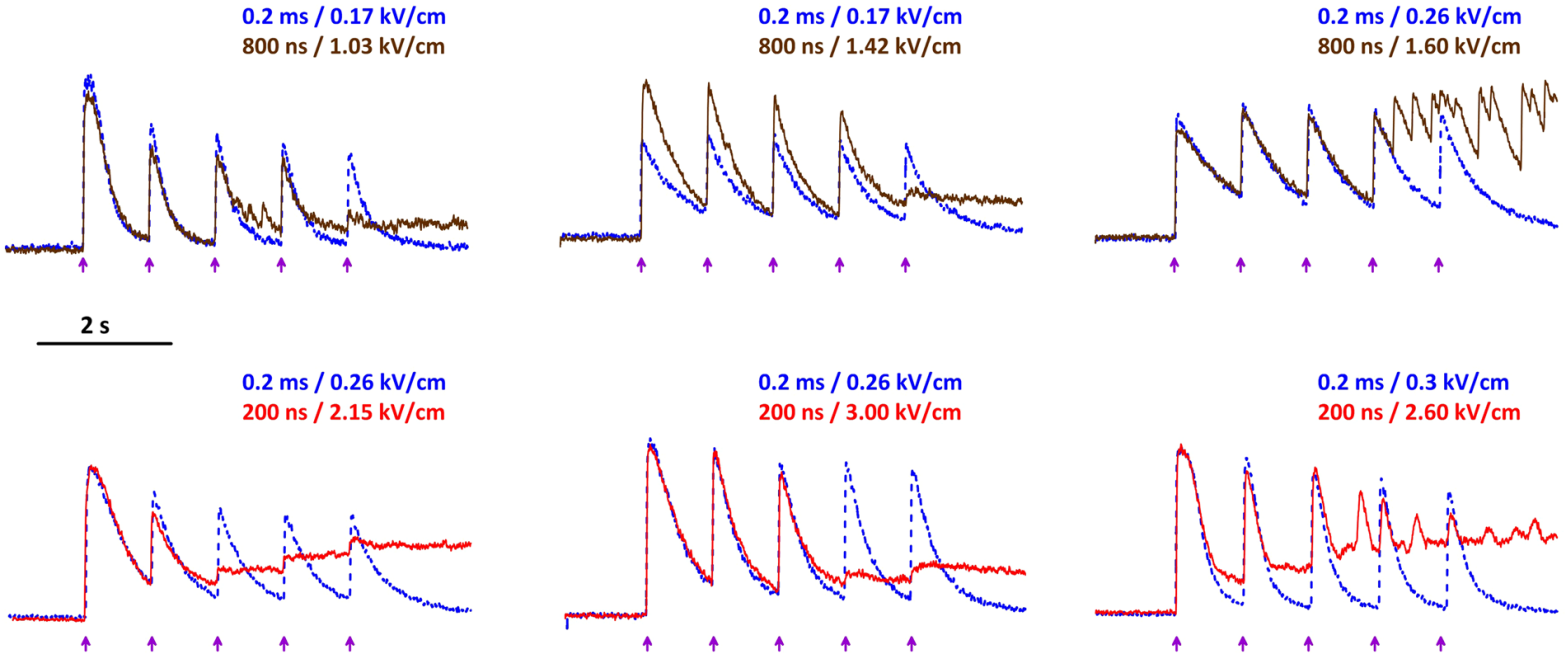
Abnormal Ca^2+^ transients in mouse VCM in response to repetitive nsPEF stimulation. Each panel is a different cell. The healthy condition of each cell was verified by its ability to respond repeatedly to conventional stimuli. A train of 5, 0.2 ms pulses at 1-s intervals (arrows) was delivered at increasing voltages until a response was detected (traces shown by a blue dotted line). This train could be applied several times, to confirm stable responses (not shown). The same procedures were repeated using 800-ns stimuli (top panels, brown solid line) or 200-ns stimuli (bottom panels, red solid line). The threshold electric field values for conventional and nsPEF stimuli are shown next to the traces.

Therefore it came out as a surprise that nsPEF performed poorly for repetitive stimulation. In most individual cells which responded reproducibly to conventional stimuli, repetitive nsPEF caused abnormal responses (Fig. 5). Cells either failed to generate one or several transients; or their shape was distorted; or cytosolic Ca^2+^ did not return to its base level. Even when the decay phase of nsPEF-induced transients was precisely the same as of conventional stimuli-induced transients (i.e., Ca^2+^ pumps were fully functional), Ca^2+^ clearance often got halted before its complete recovery to the resting level. The underlying mechanism of this phenomenon and its potential significance for defibrillation are not immediately clear, and will be explored in our future work. Of note, abnormal Ca^2+^ responses were not unique to nsPEF; they were observed with long stimuli as well, but less frequently.

### nsPEF cause less electroporative damage than conventional stimuli

A membrane-impermeable dye Pr iodide has been most frequently used to detect and quantify electroporation in cardiac myocytes^32,65–67^ and many other cell types^27,60,68,69^. Binding of propidium cation to nucleic acids inside the cell is detected by bright red fluorescence, with good resistance to bleaching. While some other dyes such as Yo-Pro-1 and cations (Tl^+^, Ca^2+^) are more sensitive for electropore detection (especially for nanopores), they are also prone to false positives due to possible entry through endogenous ion channels^18,22,27,54,70^. The larger, Pr-permeable electropores are also thought to be more injurious to the cell, resulting in lower cell survival^5^.

**Figure 6:**
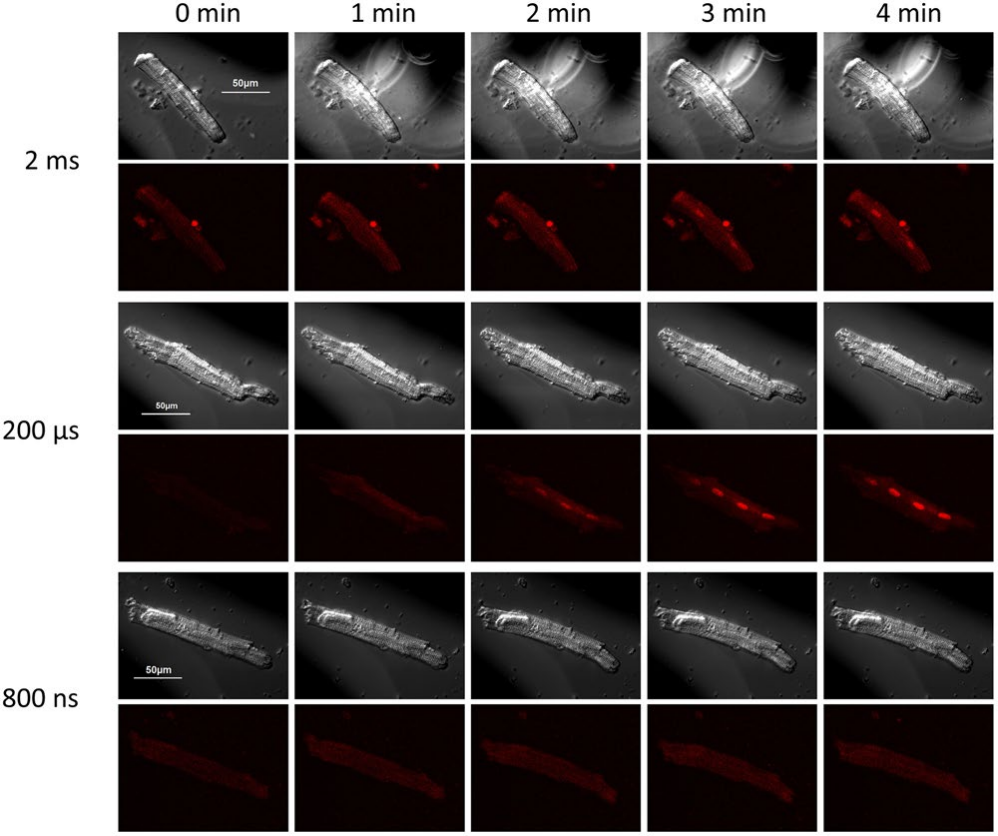
Shock duration-dependent propidium uptake in pig VCM. Shown are representative time-lapse images of 3 cells. For each cell, top row: differential interference contrast (DIC) illumination; bottom row: propidium fluorescence. The images were taken at 0, 1, 2, 3, and 4 min into experiment; a single 2-ms, 200-μs, or 800-ns shock was delivered at 27 s. The voltage of the shock was set at 5x the respective threshold for Ca^2+^ activation in each cell. Note more intense propidium uptake with longer duration shocks, and formation of large bubbles by the 2-ms stimulus.

**Figure 7:**
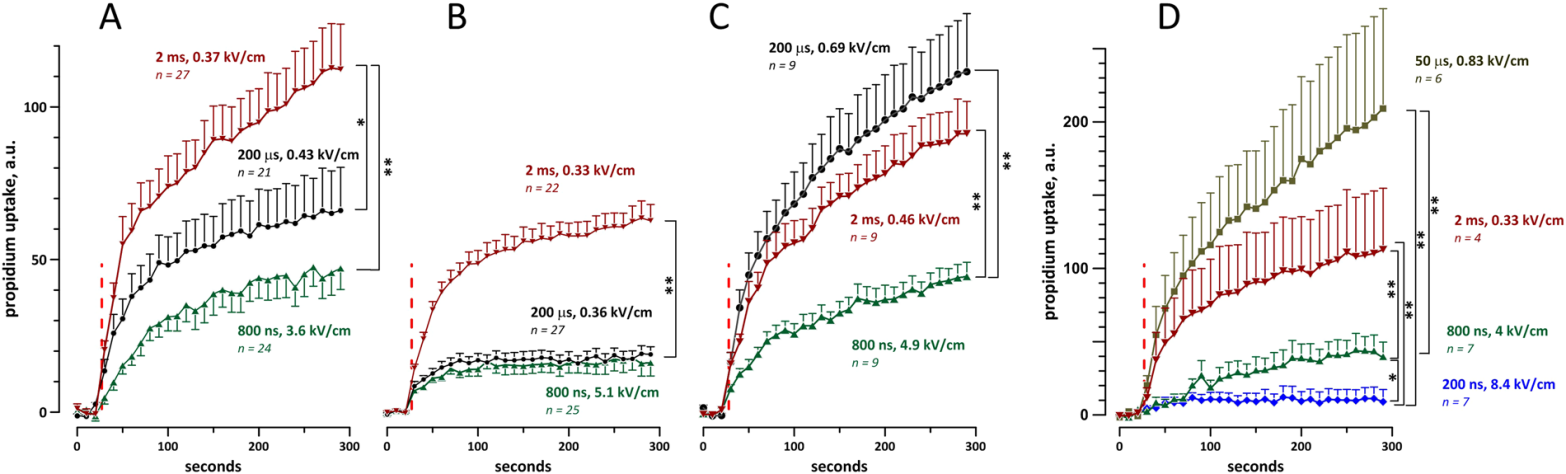
Nanosecond shocks cause less propidium uptake than longer shocks in mouse (**A**,**B**), pig (**C**), and rabbit (**D**) cardiomyocytes. In all experiments, cells were subjected to a single shock of indicated duration at 27 s into the experiment (red dashed line). The shock amplitude was set at 5x the threshold for Ca^2+^ activation in each individual cell; the respective average electric field values for each group are indicated next to the plots, along with the number of experiments in that group. Cells were oriented perpendicular to the electric field (**A**–**D**) or parallel to it (**B**). For clarity, standard error bars are shown in one direction only. *p < 0.05, **p < 0.01 with two-tailed Student’s t-test. Note that the effect of 2-ms shocks was likely reduced by bubble formation, see text for more details.

Experiments testing different shock durations were mixed in a random fashion, and only one duration was tested in any VCM. Once the excitation threshold for a given shock duration was identified, the voltage to be delivered to electrodes was increased 5-fold. Figure 6 shows examples of Pr uptake in pig VCM after a single 2-ms, 200-µs, or 800-ns shock, all delivered at 5x the excitation threshold for the respective pulse duration in each cell. Micro- and millisecond shocks consistently caused detectable Pr uptake, and, for most tested conditions, it was significantly more than with 200-ns or 800-ns pulses (Fig. 7). Of note, 2-ms pulses caused profound formation of bubbles on the surface of the cathode electrode (Fig. 6, top row), which has likely reduced the electric field “seen” by cells during the pulse, and therefore reduced the Pr uptake. Despite this reduction, 2-ms pulses at 5x threshold always caused significantly more Pr uptake than 800- or 200-ns shocks at 5x the respective thresholds.

**Figure 8:**
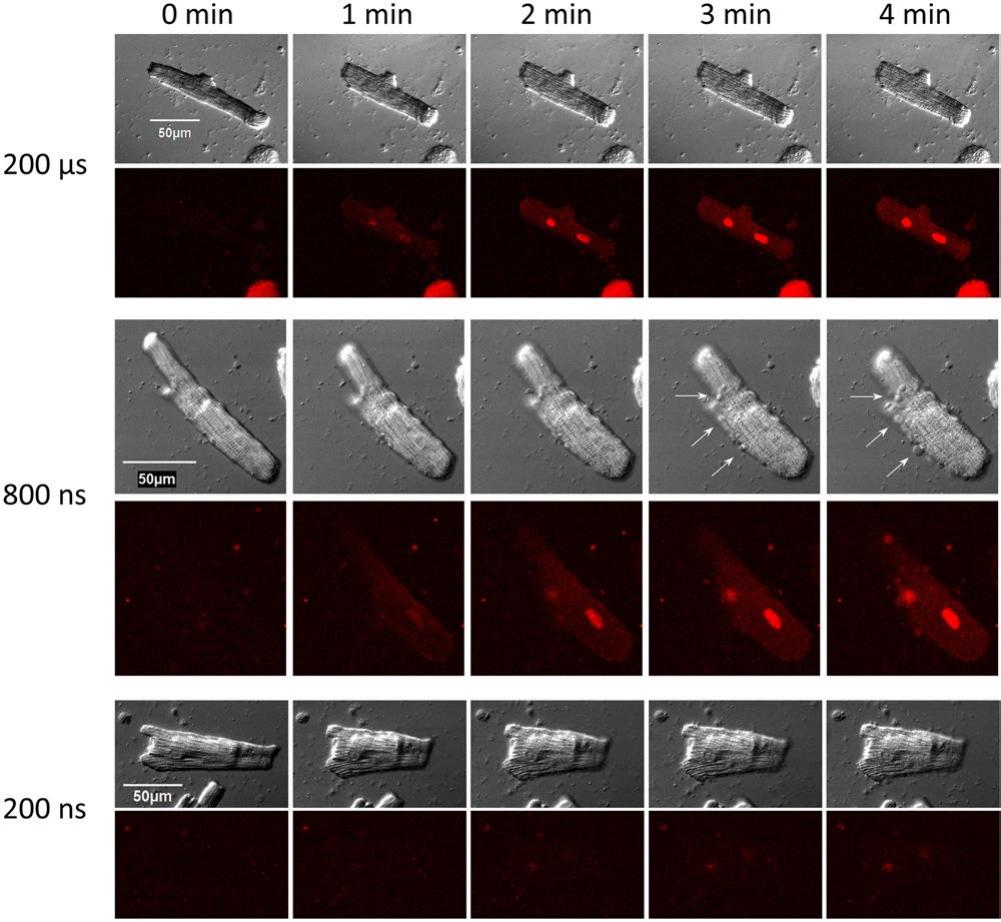
Trains of 20, 1-Hz shocks at 5x calcium activation threshold cause propidium uptake, irreversible contracture, and blebbing in mouse VCM. Trains started at 27 s into the experiment and continued for 10 s. Small blebs can be seen in DIC images at 3 and 4 min after 10-µs shocks (arrows). See Fig. 5 and text for further details.

**Figure 9:**
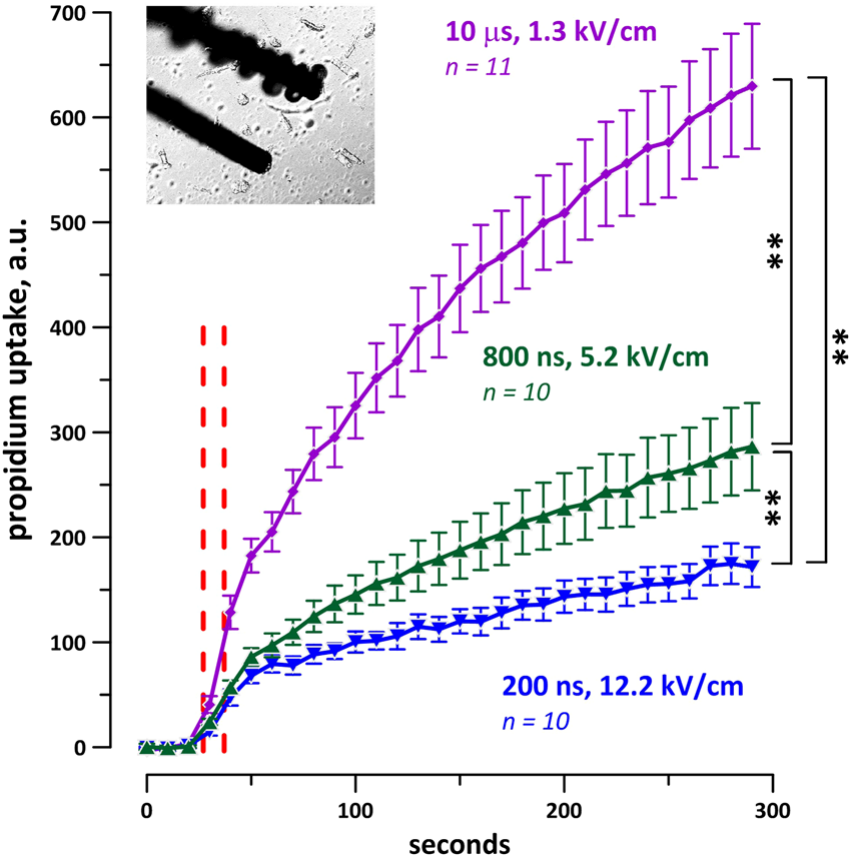
Trains of 20, 1-Hz shocks at 200 and 800 ns duration cause less Pr uptake than 10-μs shocks. Vertical dashed lines show the time interval when the shocks were applied. The shock amplitude was set at 5x calcium activation threshold for each individual cell. The inset shows the formation of gas bubbles on the cathode after a train of 20 pulses of 200-μs duration, which were therefore excluded from the analysis. See Fig. 7 and text for more details.

The correlation of Pr uptake with the pulse duration was preserved with multiple electroporating pulses (Figs 8 and 9). A train of 20 shocks (200 ns, 800 ns, 10 µs, or 200 µs duration), applied at 2 Hz and at 5x stimulation threshold, caused stable and irreversible VCM contracture, accompanied in some cells with blebbing (Fig. 8). Pr uptake was visibly similar after 200-µs shocks (Fig. 8) and 10-µs shocks (not shown); however, due to intense bubble formation on the cathode during the delivery of the pulse train (Fig. 9, inset), the 200-µs experiments were discontinued and excluded from statistics. Figure 9 shows that 200-ns shocks caused about 1.7 times less Pr uptake than 800-ns shocks (p < 0.05), and almost 4-fold less Pr uptake than 10-µs shocks (p < 0.01).

## Conclusions

This study evaluated the applicability of nanosecond electric shocks for stimulation of primary VCM from different mammalian species, and compared cell damage by shocks of different duration when the applied voltage was raised 5 times above the stimulation threshold. We found that nsPEF shocks are indeed suitable for VCM stimulation, and established the thresholds for initiation of Ca^2+^ transients in VCM from pig, mouse, and rabbit. VCM excitation is considered critical to stop propagation of fibrillation fronts, and our *in vitro* data are consistent with recent demonstration of successful nsPEF defibrillation in Langendorff-perfused rabbit hearts^12^. The reduced dependence of excitation on VCM orientation (along or across the electric field lines) will likely be beneficial in defibrillation, by enabling more uniform excitation by electric fields. At the same time, poor performance of nsPEF for repetitive stimulation of VCM indicates some additional and unknown impact, with unpredictable implications for defibrillation. As a first approximation, such effects may be related to mild nanoelectroporation of the sarcolemma and/or of the ER^18,19,22,54^, or to inhibition of voltage-gated ion channels^71,72^. In the next studies, we plan to analyze the action potentials elicited by nsPEF in VCM, in order to separate nsPEF impact on cell excitation and on downstream Ca^2+^ handling.

Exceeding the stimulation threshold 5-fold (a situation which will likely take place in at least some areas of the heart during defibrillation) caused electroporative damage, which was unambiguously manifested and quantified by Pr uptake. The extent of the damage was reduced with nsPEF, supporting earlier observations in diverse cultured cells and in embryonic VCM^5,27,73^. The freshly isolated adult VCM differ profoundly from other cell types both in cell shape and physiology, so the agreement of findings proves that the reduced formation of Pr-permeable electropores, for comparable exposure conditions, is a fundamental property of nsPEF. Although we have not evaluated here the formation of smaller, Pr-impermeable “nanoelectropores”^22,54,73^ (because it is difficult to separate them from endogenous ion channels without using channel inhibitors), the smaller pores are likely less significant for disruption of cell functions. In contrast, the presence of even a small population of larger-size pores could be a major reason for cell death^5^. Overall our findings support the idea that nsPEF shocks are a promising modality for electrostimulation and defibrillation, and set the goals for more in-depth analyses of nsPEF excitation and damage mechanisms.
